# Gut microbiota and serum metabolome in children with human metapneumovirus infection

**DOI:** 10.3389/fimmu.2026.1855983

**Published:** 2026-07-28

**Authors:** Huanqiong Li, Xiao Huang, Liyan Cai, Cuili Deng, Guangchuang Cai, Li Li, Guihua Zhang, Tiantian Liu

**Affiliations:** 1 Foshan Sanshui District People’s Hospital (Sanshui Hospital, Zhujiang Hospital, Southern Medical University), Foshan, Guangdong, China; 2 Guangdong Pharmaceutical University, Guangzhou, Guangdong, China

**Keywords:** children, gut microbiota, human metapneumovirus, metabolomics, multi-omics association

## Abstract

**Objective:**

To analyze the alterations in gut microbiota structure and serum metabolic profiles of children with human metapneumovirus (hMPV) infection using 16S rRNA high-throughput sequencing and liquid chromatography-mass spectrometry (LC-MS)-based untargeted metabolomics. Through integrated multi-omics analysis, we aim to explore the correlation between gut microbiota dysbiosis and host metabolic disorder, provide preliminary evidence for potential intervention targets, and offer primary clues for the development of preventive and therapeutic strategies including probiotic supplementation and metabolic pathway regulation.

**Methods:**

This study enrolled 42 children with hMPV infection and 12 healthy controls. Fecal samples were collected for gut microbiota analysis using 16S rRNA gene sequencing, and serum samples were obtained for metabolomic profiling via LC-MS. Various bioinformatics approaches were applied for independent analysis of microbiome and metabolome data, followed by integrated multi-omics analysis.

**Results:**

The microbial community structure of the hMPV-infected group was clearly separated from that of the healthy control group. Opportunistic pathogens such as Finegoldia, Anaerococcus, and Peptoniphilus were enriched, while beneficial commensals including Bifidobacterium, Blautia, and Faecalibacterium were reduced. Serum metabolic profiles showed global divergence between the two groups, with 558 upregulated and 213 downregulated metabolites identified. Using a VIP threshold >1.5, 24 core differential metabolites were ultimately selected. KEGG enrichment and MetPA topological analyses revealed that these differential metabolites were mainly enriched in pathways including glycerophospholipid metabolism, nucleotide metabolism, alpha-linolenic acid metabolism, and arachidonic acid metabolism. Correlation analysis showed that beneficial commensals with reduced abundance in the infected group, such as Bifidobacterium and Blautia, were positively correlated with anti-inflammatory and barrier-protective metabolites (e.g., alpha-linolenic acid, 11,12-DiHETrE, GPCho(20:4/14:0)), and negatively correlated with pro-inflammatory lipid mediators (e.g., 12,13-DiHOME, 9-OxoODE, vernolic acid).

**Conclusion:**

This study indicated that hMPV infection might be associated with intestinal microecological imbalance and serum metabolic disturbances in children. The screened core differential metabolites hold the potential for further evaluation of their diagnostic value. Significant correlations exist between gut microbiota alterations and serum metabolic disorders, providing preliminary clues for the future development of preventive and therapeutic strategies targeting gut microbiota or metabolic pathways.

## 1 Introduction

Human metapneumovirus (hMPV) is an enveloped, single-stranded, negative-sense RNA virus belonging to the family Pneumoviridae, genus Metapneumovirus, and is one of the important pathogens causing acute respiratory tract infections in children (1).Worldwide, the detection rate of hMPV ranges from approximately 5% to 15% among respiratory tract infections, and its prevalence in young children is second only to that of respiratory syncytial virus (2–9). Data from 2018 indicate that hMPV infection caused approximately 14 million cases of acute lower respiratory tract infection in children under five years of age, resulting in more than 640,000 hospitalizations and 7,700 in−hospital deaths (10). During 2024-2025, a marked surge in hMPV cases has been reported in several countries, including China, India, and the United Kingdom, further increasing the global burden and challenges of controlling respiratory infectious diseases (11). The virus was first isolated and identified from respiratory samples of children by Dutch researchers in 2001, and serological studies suggest that it has been circulating in humans for at least 50 years (7). hMPV infection is characterized by a short incubation period, high transmissibility, and the inability to induce durable protective immunity, leading to recurrent infections; young children and the elderly constitute the high−risk populations (12).

The clinical manifestations of hMPV infection are diverse. Mild cases predominantly present with upper respiratory symptoms such as nasal congestion and cough, whereas severe cases may progress to bronchitis or even pneumonia, with disease severity comparable to that of respiratory syncytial virus (RSV) infection (13). Serological surveys indicate that nearly all individuals have been infected with hMPV by five years of age (7, 14, 15). Because natural infection does not induce complete or long-lasting protective immunity, individuals may experience multiple reinfections throughout life, although clinical symptoms are generally milder than those of primary infection (16). Different genotypes (A and B) alternate in predominance every two years and frequently co-circulate, a “shift-co-circulation” pattern considered an important strategy for hMPV to evade immune pressure (17). Among children, those with underlying conditions such as congenital heart disease, bronchopulmonary dysplasia, asthma, or immunodeficiencies are at higher risk of developing severe infection (18, 19).

Currently, no specific antiviral therapy is available for hMPV infection, and clinical management remains primarily supportive, including oxygen therapy and mechanical ventilation (20). In terms of pharmacotherapy, ribavirin, immunoglobulins, and empirical antibiotics may be used in certain clinical settings (21). Vaccine development represents a key direction for hMPV prevention and control. Although inactivated vaccines, live attenuated vaccines, and F protein subunit vaccines have been validated in animal models, none have yet entered human clinical trials (20). However, animals vaccinated with formalin-inactivated hMPV exhibited exacerbated clinical symptoms upon subsequent wild-type hMPV infection, which may be associated with enhanced pulmonary pathology and dysregulated immune responses (22). The incomplete and short-lived protective immunity induced by natural infection, together with the risk of vaccine-associated enhanced disease, poses multiple challenges for hMPV vaccine development (23, 24).

In recent years, multi-omics technologies have provided new perspectives for research on infectious diseases. The gut microbiota, often referred to as the “second genome of humans,” participates in immune homeostasis regulation through the “gut-lung axis” (25–27).Studies have shown that influenza virus infection can alter the composition of the gut microbiota, and microbiota dysbiosis in turn impairs host antiviral immunity (28). The gut microbiota characteristics of children with RSV infection are associated with disease severity (29). Patients with SARS-CoV-2 infection commonly exhibit significant gut microbiota dysbiosis, and the degree of dysbiosis is positively correlated with disease severity and inflammatory levels (30). However, evidence regarding the impact of hMPV infection on the gut microbiota remains lacking. Metabolomics can directly reflect endogenous metabolic information, and the serum metabolic profile serves as a window that integrates metabolic changes across various tissues and organs (31). Viral infection typically induces adaptive alterations in host immune metabolism, with significant perturbations observed in energy, lipid, and amino acid metabolic pathways (32). Such metabolic alterations are common in various viral infections, including influenza, dengue fever, and COVID-19, and certain metabolic features may serve as potential diagnostic or prognostic biomarkers (33–35).Nevertheless, the serum metabolic characteristics of hMPV infection remain unclear.

Based on this background, the present study employed 16S rRNA high-throughput sequencing and liquid chromatography-mass spectrometry (LC-MS)-based untargeted metabolomics to analyze alterations in the gut microbiota structure and serum metabolic profiles of children with hMPV infection. Through integrated multi-omics analysis, we aimed to explore the association between gut microbiota dysbiosis and host metabolic dysregulation, and to screen potential intervention targets, thereby providing a theoretical basis for the future development of novel prevention and treatment strategies such as probiotic supplementation and metabolic pathway modulation.

## 2 Methods

### 2.1 Study participants

All eligible subjects or their legal guardians provided written informed consent. From January 1 to May 31, 2025, a total of 42 hospitalized patients with human metapneumovirus (hMPV) infection (hereinafter referred to as the EG group) were enrolled in this study, all of whom were children aged ≤14 years. The diagnosis of hMPV infection was based on the diagnostic guidelines of the Chinese Medical Association and the World Health Organization. Clinical data, including age, sex, cough, fever, white blood cell count, lymphocyte count, and etiological information, were extracted from the hospital electronic medical record system. In addition, 12 age-matched healthy volunteers without acute infection were recruited as the control group (hereinafter referred to as the CG group).

### 2.2 Sample collection

Fecal samples were collected via rectal swab within 24–48 hours after admission. Before sampling, the subjects fasted for 8 hours (36), and the perianal area was thoroughly cleaned with soap, clean water, and 75% alcohol. Importantly, the alcohol was applied exclusively to the external perianal skin and did not contact the anal canal or rectal mucosa, as the anal sphincter remained closed during the cleaning procedure. After alcohol evaporation, A sterile swab was inserted approximately 4–5 cm into the anus and gently rotated to obtain the fecal sample. The swab was immediately placed in a sterile tube and stored at −80°C. Whole blood was collected in vacuum blood collection tubes and allowed to stand at 4°C for 30 to 120 minutes, avoiding vigorous shaking to prevent hemolysis. The blood samples were then centrifuged at 3,000 rpm for 10 minutes at 4°C. The upper serum layer was collected, and protease inhibitors were added. The serum was aliquoted into 0.5 mL centrifuge tubes (150 μL per tube), snap-frozen in liquid nitrogen for 30 seconds, and subsequently transferred to −80°C for storage.

### 2.3 Inclusion and exclusion criteria for study subjects

For the disease group, eligible participants were hospitalized children aged 14 years or younger with clinically diagnosed and laboratory-confirmed hMPV infection whose guardians provided voluntary written informed consent after comprehension of the study objectives, procedures and sample utilization, and who were accessible for anal swab and fasting venous blood collection within 24 to 48 hours upon admission; patients were excluded if they had taken systemic antibiotics, antifungals, probiotics or prebiotics four weeks prior to enrollment, suffered from chronic disorders interfering with gut microbiota or systemic metabolism, had congenital or acquired immunodeficiency or received immunosuppressive treatment throughout the enrollment phase, or presented with active intestinal or systemic infections induced by other pathogens at enrollment. Healthy children matched with the disease group in age, gender, feeding pattern and residential region were enrolled as controls, who had no recent respiratory or gastrointestinal infectious symptoms, displayed normal physical examination results, and whose guardians provided signed written informed consent. Children were excluded from the control group if they had taken antibiotics, antifungal agents, probiotics or prebiotics within the previous four weeks, or suffered from immune dysfunction, chronic underlying diseases or congenital developmental abnormalities.

### 2.4 The 16S rRNA gene sequence analysis

Total DNA was extracted from anal swab samples using the FastPure Stool DNA Isolation Kit (magnetic bead method). The purity, concentration, and integrity of the extracted DNA were assessed using a NanoDrop 2000 spectrophotometer and 1% agarose gel electrophoresis. The V3–V4 hypervariable region of the 16S rRNA gene was amplified by PCR using primers 338F and 806R (37). The PCR products were verified by 2% agarose gel electrophoresis, purified using a magnetic bead method, and quantified and normalized using a Synergy HTX microplate reader. Amplification region and primers: The V3–V4 hypervariable region of the 16S rRNA gene was amplified using forward primer 338F (5’-ACTCCTACGGGAGGCAGCAG-3’) and reverse primer 806R (5’-GGACTACHVGGGTWTCTAAT-3’).PCR reaction mixture (20 μL): 4 μL of 5×FastPfu Buffer, 2 μL of 2.5 mM dNTPs, 0.8 μL of forward primer (5 μM), 0.8 μL of reverse primer (5 μM), 0.4 μL of FastPfu DNA Polymerase, 0.2 μL of BSA, 10 ng of template genomic DNA, and sterile deionized water added to a final volume of 20 μL.Amplification program: Initial denaturation at 95°C for 3 min, followed by 30 cycles of denaturation at 95°C for 30 s, annealing at 60°C for 30 s, and extension at 72°C for 45 s. After the cycles, a final extension at 72°C for 10 min, followed by holding at 10°C.Polymerase: FastPfu DNA Polymerase; total number of cycles: 30; expected amplicon length: approximately 468 bp based on the primer target region. A paired-end (PE) library was constructed using the NEXTFLEX^®^ Rapid DNA-Seq Kit, and high-throughput sequencing was performed on the Illumina NextSeq 2000 PE300 platform.

Raw sequencing data were quality−filtered using fastp (38) and merged using FLASH (39). Subsequent analyses were carried out using the Qiime2 pipeline (40). Amplicon sequence variants (ASVs) were generated via the DADA2 plugin (41) after removing chloroplast and mitochondrial sequences, and all samples were rarefied to 20,000 sequences per sample. Taxonomic annotation was performed based on the SILVA 16S rRNA database (version 138.2). Subsequent data analyses were conducted on the Majorbio Cloud Platform. Alpha diversity indices for each group were calculated using mothur software (42). and intergroup differences were assessed using the Wilcoxon rank−sum test. Differential microbial taxa between groups were identified using the Wilcoxon rank-sum test. To account for multiple comparisons, the Benjamini-Hochberg false discovery rate (FDR) correction was applied, and taxa with an FDR-adjusted q-value < 0.10 were considered significantly different. For community structure similarity, principal coordinate analysis (PCoA) was performed based on the Bray−Curtis distance algorithm, and the statistical significance of differences in community structure between groups was evaluated using permutational multivariate analysis of variance (PERMANOVA). Finally, linear discriminant analysis effect size (LEfSe) analysis (43) was used to identify bacterial taxa with significant differences between groups from the phylum to the genus level. A species correlation network was constructed using Spearman correlation coefficients with thresholds of |r| > 0.5 and P < 0.05 (44).

### 2.5 Plasma lipid metabolite analysis based on UHPLC-MS/MS

An aliquot of 100 μL of each serum sample was precisely transferred, followed by the addition of 300 μL of a methanol:acetonitrile (1:1, v/v) extraction solution containing four internal standards including L-2-Chlorophenylalanine,Palmitoyl-L-carnitine-(N-methyl-d3), Cholic acid-2,2,4,4-d4 and L-Phenylalanine-d5. The mixture was vortexed and subjected to low-temperature ultrasonic extraction at 5°C and 40 kHz for 30 min, then allowed to stand at −20°C for 30 min. After centrifugation at 13,000 × g for 15 min at 4°C, the supernatant was collected and dried under a nitrogen stream. The residue was reconstituted with 100 μL of acetonitrile:water (1:1, v/v), vortexed, subjected to low-temperature ultrasonic extraction, and centrifuged again. The resulting supernatant was transferred into an autosampler vial with an insert for analysis. Quality control (QC) samples were prepared by pooling 20 μL of supernatant from each sample and processed using the same protocol. QC samples were inserted after every 5-15 experimental samples throughout the analytical run to monitor instrument stability and data reproducibility.

LC-MS analysis was performed using a Thermo Fisher UHPLC-Exploris 240 system equipped with an ACQUITY UPLC HSS T3 column (100 mm × 2.1 mm i.d., 1.8 μm). Mobile phase A consisted of 95% water + 5% acetonitrile containing 0.1% formic acid, and mobile phase B consisted of 47.5% acetonitrile + 47.5% isopropanol + 5% water containing 0.1% formic acid. The injection volume was 3 μL, and the column temperature was maintained at 40°C. Mass spectrometry was performed using electrospray ionization (ESI) in both positive and negative ion modes. The scan range was m/z 70–1050. The sheath gas flow rate was set to 60 arb, auxiliary gas flow rate to 20 arb, and heating temperature to 350°C. The capillary temperature was 320°C. Spray voltages were set to 3400 V (positive mode) and −3000 V (negative mode), with an S−Lens RF level of 70. Normalized collision energies were set to 20%, 40%, and 60%. The resolution for full MS and MS² was 60,000 and 15,000, respectively.

Raw data were processed using Progenesis QI software for baseline filtering, peak detection, integration, retention time correction, and alignment. Metabolite identification and annotation were performed by matching against the HMDB, Metlin, and Majorbio in−house databases. The resulting data matrix was uploaded to the Majorbio Cloud Platform for preprocessing. After filtering based on the 80% rule, filling missing values with the minimum value, total sum normalization, and removing variables with QC sample RSD > 30%, log10 transformation was applied. Principal component analysis (PCA) and orthogonal partial least squares discriminant analysis (OPLS-DA) were performed using the R package “ropls” (version 1.6.2). Model stability was evaluated by 7−fold cross−validation. Differential metabolites were selected based on a variable importance in projection (VIP) threshold > 1.5 and a P value < 0.05 (Student’s t−test). Metabolic pathway annotation was performed using the KEGG database (https://www.kegg.jp/kegg/pathway.html). Pathway enrichment analysis was conducted using Fisher’s exact test implemented in the Python scipy.stats package. KEGG pathway enrichment analysis was performed using Fisher’s exact test, followed by Benjamini-Hochberg FDR correction. Pathways with a q-value < 0.10 were considered significantly enriched

### 2.6 Correlation analysis

Spearman’s rank correlation coefficients were calculated to quantify pairwise associations between gut microbial genera and serum metabolites within the study cohort, and the resulting correlation matrix was visualized via heatmaps. For associations between differential gut taxa, differential metabolites and four core clinical indicators (length of hospital stay, C-reactive protein [CRP], interleukin-6 [IL-6], procalcitonin [PCT]), Spearman’s correlation analysis was also adopted to systematically assess correlative relationships, with the outcomes displayed as a correlation heatmap. In addition, Pearson correlation coefficients were used to quantify the correlation between metabolites and gut microbiota among samples in the same group; an absolute correlation coefficient closer to 1 represents a stronger correlative relationship between the two variables.

## 3 Results

### 3.1 Clinical baseline

A total of 42 hospitalized children with hMPV infection were enrolled in this study, and their demographic and clinical baseline data are summarized as follows. Regarding demographic distribution, there were 20 males (47.62%) and 22 females (52.38%), indicating a balanced sex distribution. The majority of patients were infants and young children aged ≥29 days to ≤3 years (22 cases, 52.38%). In terms of clinical manifestations, all children presented with fever and cough (42 cases, 100% each). Other common symptoms included rhinorrhea (36 cases, 85.71%) and sputum production (33 cases, 78.57%). Abnormal lung auscultation findings were observed in 32 cases (76.19%), and chest imaging indicated pulmonary infection in 38 cases (90.48%). The length of hospital stay was concentrated in the range of 3–5 days (33 cases, 78.57%), suggesting a favorable treatment response. Laboratory examination showed that routine blood parameters were generally within normal ranges. Among inflammatory markers, procalcitonin was elevated in 40 cases (95.24%), and interleukin−6 was elevated in 34 cases (80.95%). Detailed clinical data are presented in **Supplementary Tables 1** and **2**.

### 3.2 Gut microbiota characteristics

Following quality filtering, a total of 1,111,860 high-quality sequences were obtained from 54 samples, with an average of 20,590 sequences per sample. A total of 2,844 ASVs were identified, averaging 53 ASVs per sample (**Supplementary Table 3**). Rarefaction curves for both richness (Sobs) and diversity (Shannon index) approached a plateau, indicating that the sequencing depth adequately captured the microbial diversity in the samples (**Supplementary Figure 1**).

**Figure 1:**
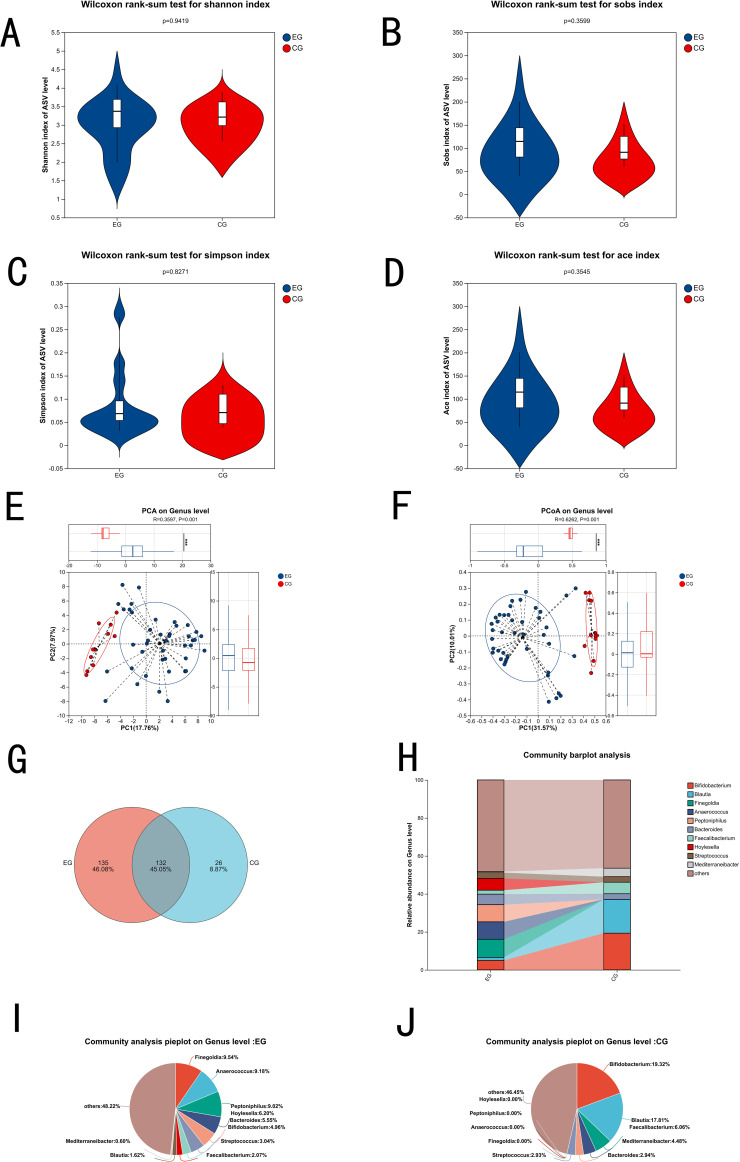
Analysis of gut microbiota alpha diversity, beta diversity, and community composition in children with hMPV infection (EG group) and healthy controls (CG group). **(A–D)** Violin plots of alpha diversity indices (Shannon, Sobs, Simpson, Ace) analyzed by Wilcoxon rank-sum test. **(E)** PCA and **(F)** PCoA plots at the genus level showing beta diversity between groups**. (G)** Venn diagram of shared and unique ASVs in EG and CG groups. **(H)** Genus-level community barplot analysis; **(I, J)** Pie plots of genus-level distribution in EG and CG groups, respectively.

The alpha diversity indices were compared between the EG and CG groups using the Wilcoxon rank-sum test. No statistically significant differences were observed in the Sobs, Shannon, ACE, or Simpson indices between the two groups, indicating that the gut microbiota of the EG and CG groups were similar in terms of species richness and community structural complexity (**Figures 1A–D**). In contrast, beta diversity analysis revealed a clear separation of microbial community structures between the two groups. Both principal component analysis (PCA) and principal coordinate analysis (PCoA) showed distinct clustering of samples from the EG and CG groups. Subsequent intergroup difference analysis (ANOSIM) further confirmed that the overall structure of the gut microbiota differed significantly between the two groups (PCA: R = 0.5145, P = 0.001; PCoA: R = 0.7324, P = 0.001) (**Figures 1E, F**).

The Venn diagram illustrated the distribution characteristics of bacterial genera between the two groups. A total of 267 genera were identified in the EG group, compared with 158 genera in the CG group. Among these, 135 genera were uniquely present in the EG group, while 23 genera were uniquely present in the CG group (**Figure 1G**).

Taxonomic analysis revealed the species composition at different classification levels between the two groups. At the phylum level, the EG group was predominantly composed of *Bacillota* (60%), *Bacteroidota* (21.39%), *Actinomycetota* (11.42%), *Pseudomonadota* (3.45%), and *Campylobacterota* (1.74%). In contrast, the CG group was mainly composed of *Bacillota* (67.9%), *Actinomycetota* (23.17%), *Bacteroidota* (4.5%), and *Pseudomonadota* (4.28%) (**Supplementary Figures 2A–C**). At the genus level, the dominant genera in the EG group were *Finegoldia* (9.54%), *Anaerococcus* (9.18%), *Peptoniphilus* (9.02%), *Hoylesella* (6.2%), *Bacteroides* (5.55%), *Bifidobacterium* (4.96%), *Streptococcus* (3.04%), *Faecalibacterium* (2.07%), *Blautia* (1.62%), and *Mediterraneibacter* (0.6%). In the CG group, the dominant genera were *Bifidobacterium* (19.32%), *Blautia* (17.81%), *Faecalibacterium* (6.06%), *Mediterraneibacter* (4.48%), *Bacteroides* (2.94%), and *Streptococcus* (2.93%) (**Figures 1H–J**).

**Figure 2:**
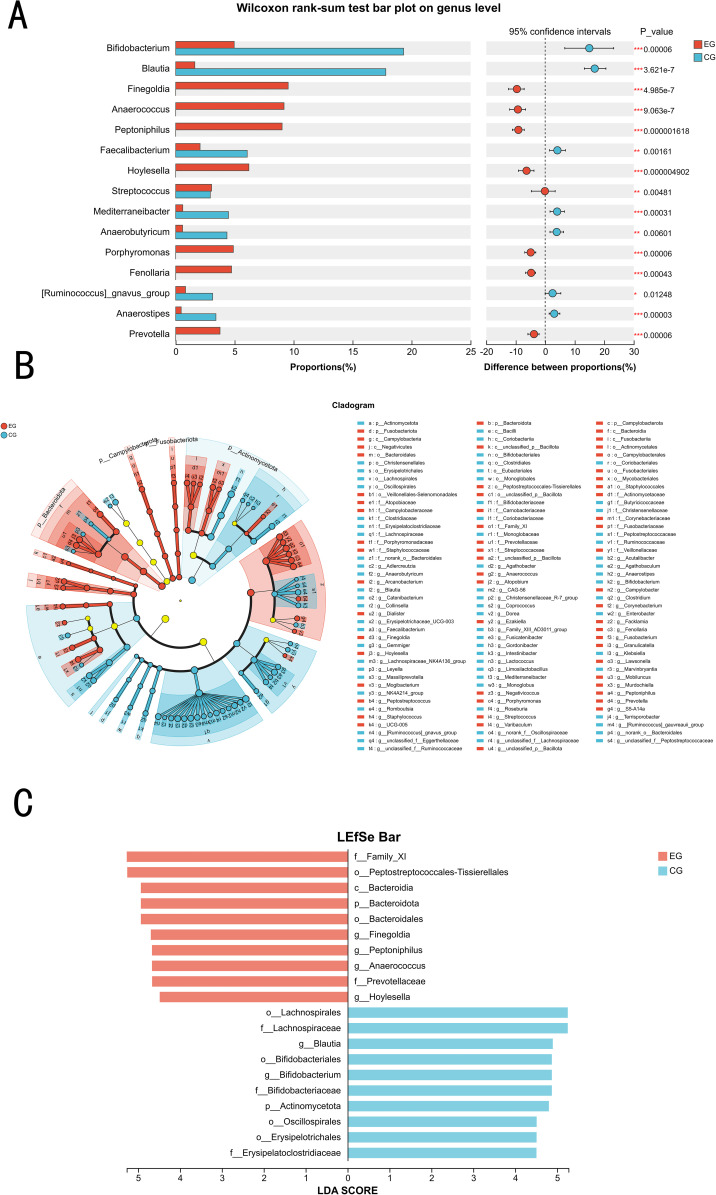
Identification of differential gut microbiota between children with hMPV infection (EG group) and healthy controls (CG group). **(A)** Wilcoxon rank-sum test bar plot showing significantly different genera between the two groups, with 95% confidence intervals and P-values indicated. **(B)** LEfSe cladogram illustrating the taxonomic hierarchy of differential microbial taxa. **(C)** LEfSe bar plot displaying the linear discriminant analysis (LDA) scores of key differential taxa, highlighting biomarkers enriched in EG (red) and CG (blue) groups.

Wilcoxon rank-sum tests were performed to identify species with significantly different abundances between the two groups. At the phylum level, the abundance of *Actinomycetota* was significantly higher in the CG group than in the EG group, whereas the abundances of *Bacteroidota*, *Campylobacterota*, and *Fusobacteriota* were significantly higher in the EG group than in the CG group (**Supplementary Figure 2D**). At the genus level, the abundances of *Bifidobacterium*, *Blautia*, *Faecalibacterium*, *Mediterraneibacter*, and *Anaerobutyrium* were significantly higher in the CG group than in the EG group, while the abundances of *Finegoldia*, *Anaerococcus*, *Peptoniphilus*, *Hoylesella*, and *Streptococcus* were significantly higher in the EG group than in the CG group (**Figure 2A**).

To further identify potential biomarkers, LEfSe analysis was performed with a linear discriminant analysis (LDA) score threshold ≥2 (**Figures 2B, C**). The EG group was characterized by enrichment of *Family_XI*, *order Peptostreptococcales-Tissierellales*, *class Bacteroidia*, *phylum Bacteroidota*, *order Bacteroidales*, *genus Anaerococcus*, *genus Finegoldia*, *genus Peptoniphilus*, *family Prevotellaceae*, and *genus Hoylesella*. In contrast, the CG group exhibited enrichment of *order Lachnospirales*, *family Lachnospiraceae*, *genus Blautia*, *order Bifidobacteriales*, *genus Bifidobacterium*, *family Bifidobacteriaceae*, *order Oscillospirales*, *family Ruminococcaceae*, *order Erysipelotrichales*, and *family Erysipelatoclostridiaceae*.

### 3.3 Untargeted metabolomics results

**Figure 3:**
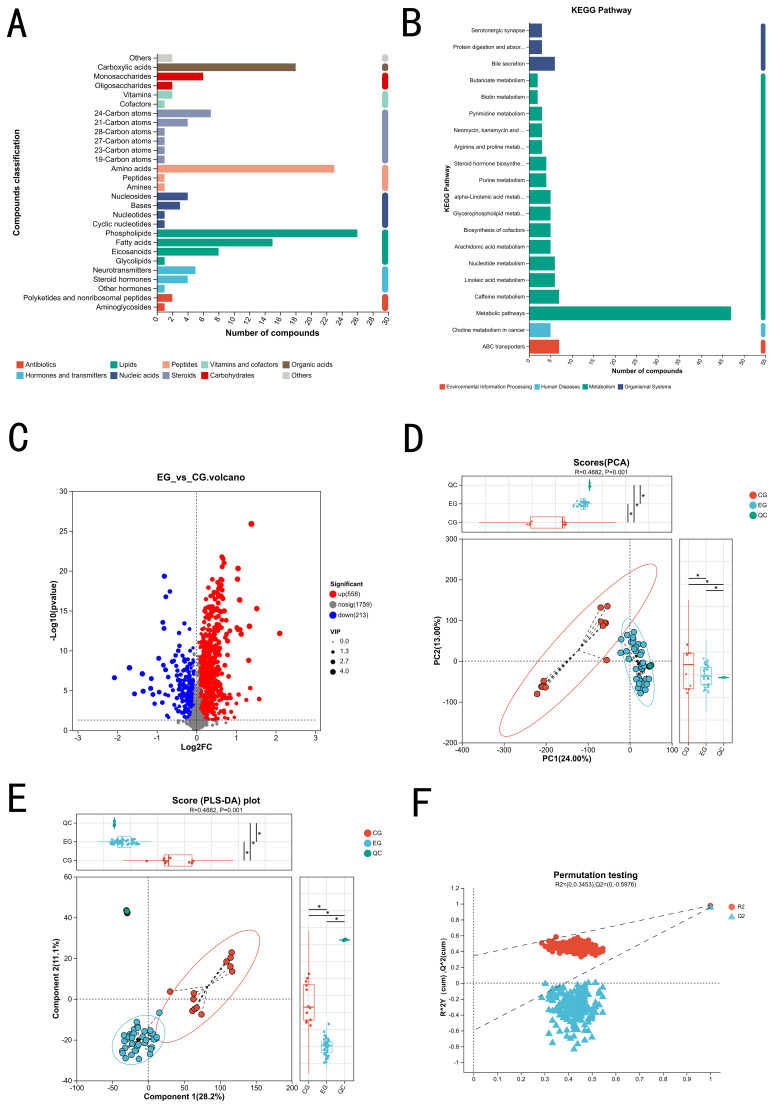
Serum untargeted metabolomic profiling and functional analysis in children with hMPV infection (EG group) and healthy controls (CG group). **(A)** Classification of identified serum metabolites by compound type. **(B)** KEGG pathway enrichment analysis of differential metabolites. **(C)** Volcano plot displaying significantly differential metabolites between EG and CG groups. **(D)** PCA score plot, **(E)** PLS-DA score plot, and **(F)** permutation testing plot showing the separation of metabolic profiles between the two groups.

The identified metabolites were classified according to their chemical taxonomy. Lipids accounted for the highest proportion (34.97%), followed by Peptides (17.48%) and Organic acids (12.59%) (**Figure 3A**). KEGG analysis indicated that the synthesis of these metabolites is regulated by various metabolic pathways, including Metabolic pathways, Linoleic acid metabolism, Nucleotide metabolism, and Glycerophospholipid metabolism (**Figure 3B**).

Using the criteria of fold change > 1 (upregulated) or < 1 (downregulated) and P < 0.05, a total of 558 upregulated and 213 downregulated metabolites were identified. The volcano plot intuitively shows the overall distribution of the upregulated and downregulated metabolites (**Figure 3C**). Notably, 558 metabolites were significantly upregulated, including pregnenolone sulfate, galactosylglycerol, S−(formylmethyl)glutathione, and isobutyric acid, while 213 metabolites were significantly downregulated, including alpha−linolenic acid and 4′−phosphopantothenoylcysteine.

Principal component analysis (PCA) showed that samples from the EG and CG groups were clearly separated along the first principal component axis, indicating a global difference in metabolic profiles between the two groups. Samples in the EG group clustered tightly (small intragroup variation), whereas those in the CG group were more dispersed (higher intragroup heterogeneity). Quality control (QC) samples were highly clustered, suggesting good instrument stability and reproducibility (**Figure 3D**). To further analyze the metabolic differences between the two groups, orthogonal partial least squares discriminant analysis (OPLS−DA) was performed. As shown in **Figure 3E**, the two groups were clearly separated on the OPLS−DA score plot. To further validate model reliability and avoid overfitting inherent to supervised analysis, a permutation test was conducted. As illustrated in **Figure 3F**, both R² (model interpretability) and Q² (model predictability) decreased with decreasing permutation retention, confirming that the model was robust and valid.

**Figure 4:**
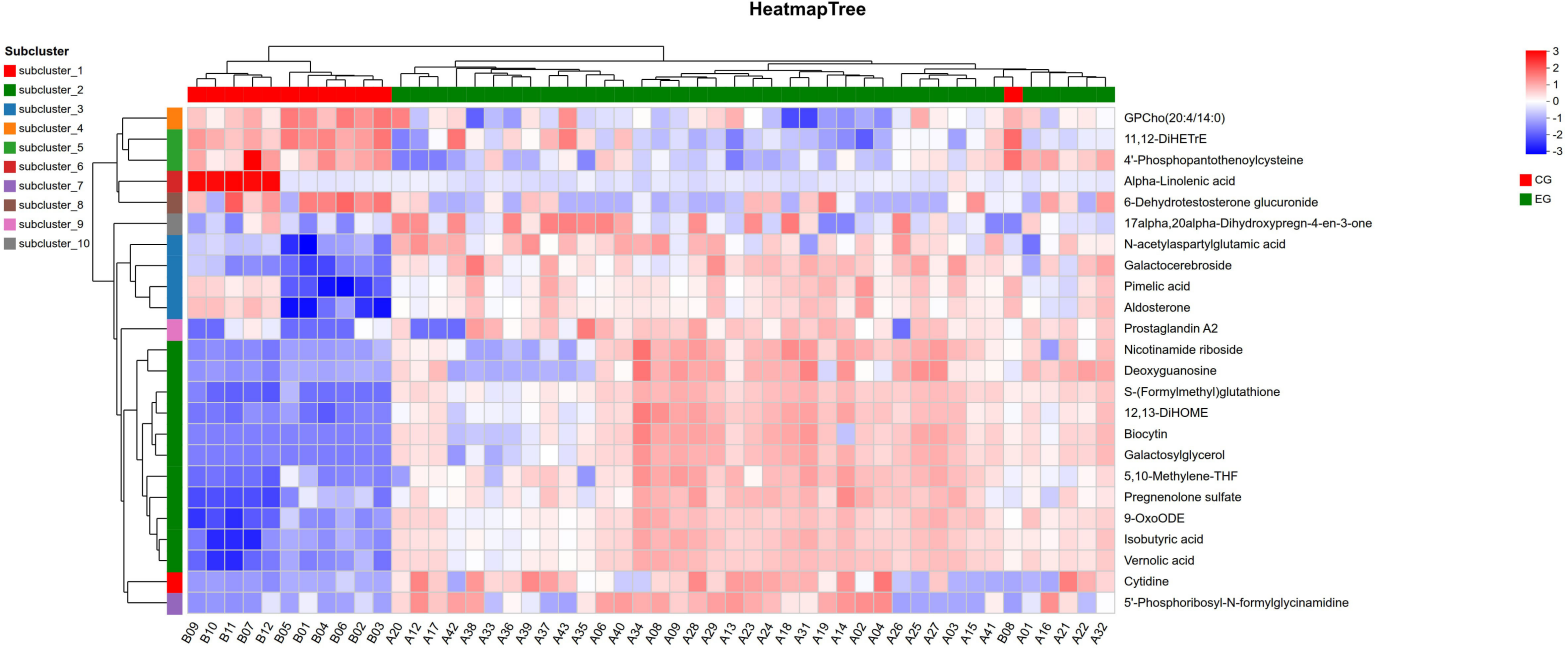
HeatmapTree analysis of gut microbiota and serum metabolites in hMPV-infected (EG) and healthy control (CG) children. The heatmap shows the relative abundance of key taxa and metabolites, with hierarchical clustering indicating sample separation.

Hierarchical clustering heatmap (**Figure 4**) completely separated the samples of the EG group from those of the CG group, further confirming a globally significant difference in serum metabolic profiles between the two groups. The differential metabolites exhibited a polarized expression pattern: pro−inflammatory metabolites were generally upregulated in the infected group, whereas metabolites related to anti−inflammation and host homeostasis maintenance were significantly downregulated, directly reflecting the host metabolic dysregulation induced by hMPV infection.

**Figure 5:**
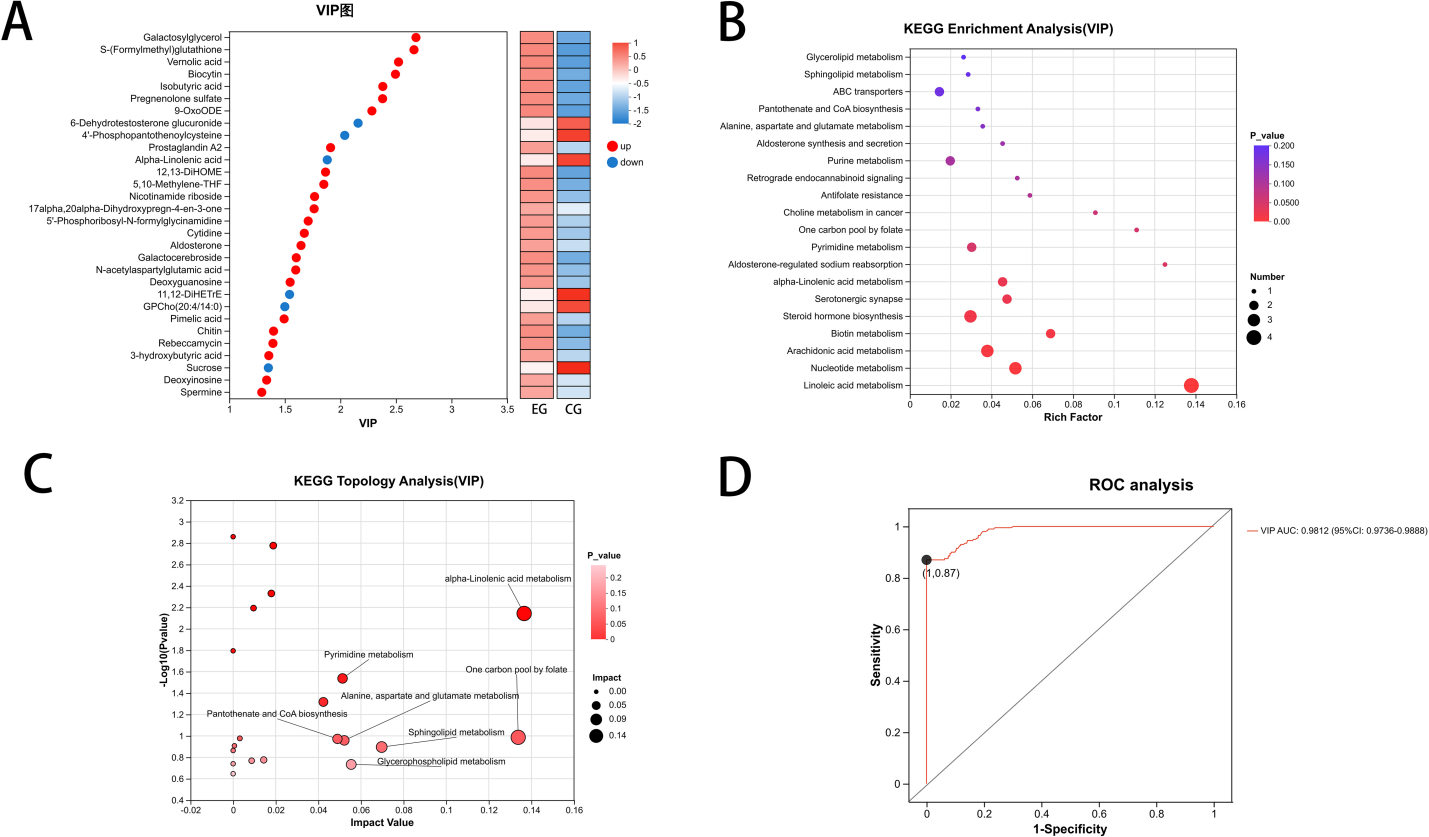
**(A)** Integrated analysis of serum metabolic functions and construction of a diagnostic model for pediatric hMPV infection. **(A)** Variable Importance in the Projection (VIP) plot from orthogonal partial least squares discriminant analysis (OPLS-DA) showing key differential metabolites. **(B)** KEGG pathway enrichment analysis and **(C)** corresponding topology analysis mapping the biological processes and pathways impacted by hMPV infection. **(D)** Receiver Operating Characteristic (ROC) curve evaluating the diagnostic performance of the core metabolic signature for hMPV infection.

Using a threshold of VIP > 1.5, a total of 24 key differential metabolites were identified between the EG and CG groups (**Supplementary Table 4**). The bubble plot (**Figure 5A**) showed that the upregulated metabolites included galactosylglycerol, S−(formylmethyl)glutathione, vernolic acid, biocytin, isobutyric acid, pregnenolone sulfate, and 9−OxoODE, while the downregulated metabolites included 6−dehydrotestosterone glucuronide, 4′−phosphopantothenoylcysteine, GPCho(20:4/14/0), 11,12−DiHETrE, and alpha−linolenic acid.

KEGG pathway enrichment analysis (**Figure 5B**) revealed that the significantly differential metabolites were mainly enriched in pathways including linoleic acid metabolism, nucleotide metabolism, arachidonic acid metabolism, biotin metabolism, steroid hormone biosynthesis, serotonergic synapse, alpha−linolenic acid metabolism, protein digestion and absorption, and glycerophospholipid metabolism. MetPA analysis (**Figure 5C**) further identified pathways that serve as key nodes in the global metabolic network and exhibited high enrichment significance, including nucleotide metabolism, alpha−linolenic acid metabolism, pyrimidine metabolism, purine metabolism, sphingolipid metabolism, and glycerophospholipid metabolism.

Based on the 24 differential metabolites with VIP > 1.5, ROC analysis was performed to screen metabolites with AUC ≥ 0.9 as potential biomarkers. The results showed that S−(formylmethyl)glutathione, vernolic acid, 12,13−DiHOME, biocytin, isobutyric acid, and chitin exhibited good predictive performance (**Supplementary Table 5**). The combined diagnostic model achieved an AUC of 0.9812 (95% CI: 0.9736–0.9888), indicating a high discriminative value for pediatric hMPV infection (**Figure 5D**).

### 3.4 Correlation analysis

**Figure 6:**
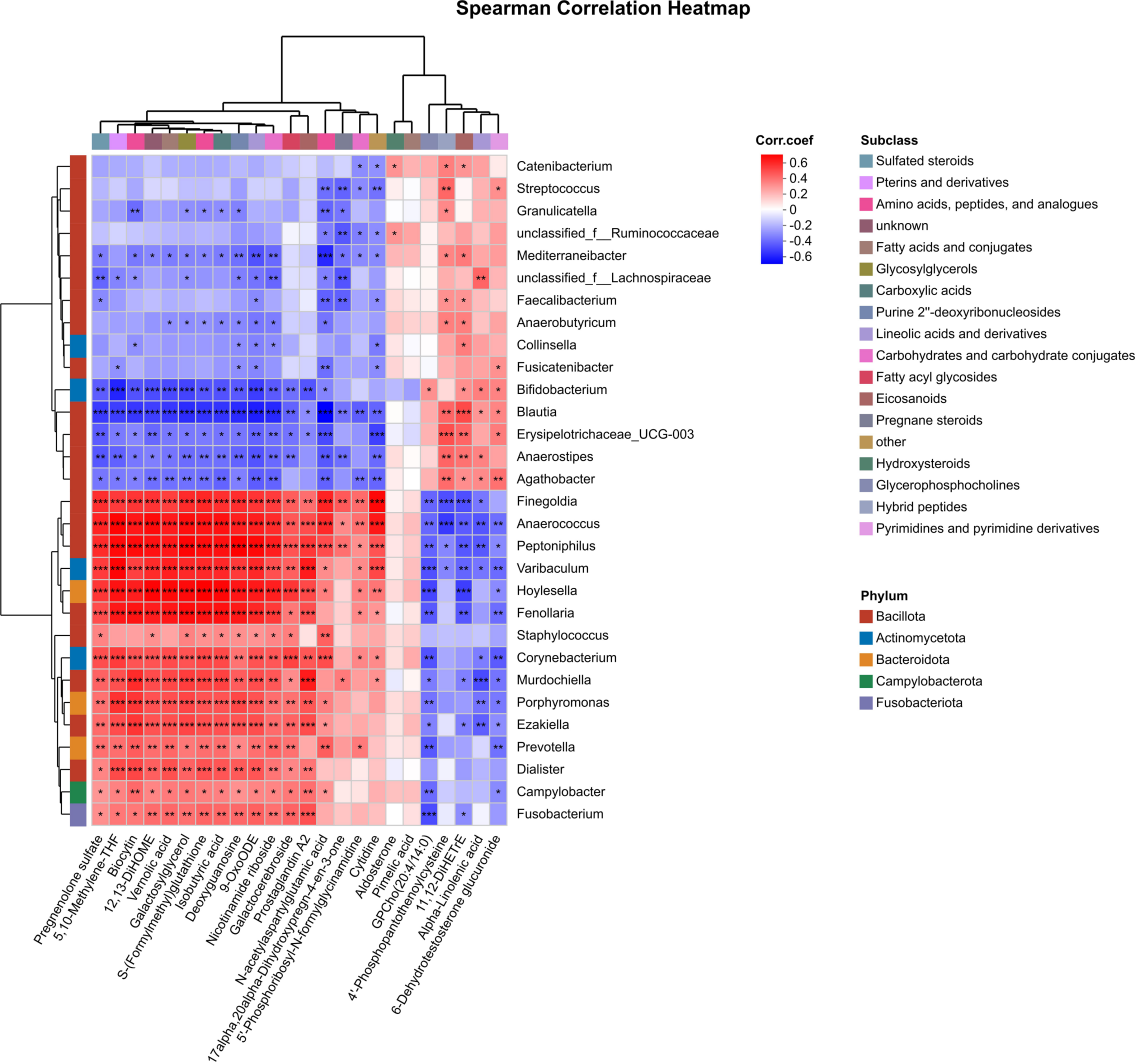
Spearman correlation heatmap analysis of gut microbiota genera and serum metabolites in children with hMPV infection, revealing significant microbiota-metabolite interactions.

Correlation analysis revealed that in the hMPV-infected group, opportunistic pathogens with increased abundance (e.g., *Finegoldia*, *Anaerococcus*, *Peptoniphilus*, *Varibaculum*, *Hoylesella*, *Fenollaria*, *Campylobacter*, *Fusobacterium*) were significantly positively correlated with pro-inflammatory lipid mediators (prostaglandin A2, 12,13-DiHOME, vernolic acid, 9-OxoODE) and oxidative stress markers (S−(formylmethyl)glutathione, isobutyric acid, nicotinamide riboside), while being significantly negatively correlated with potentially anti-inflammatory metabolites (11,12-DiHETrE, GPCho(20:4/14/0), alpha−linolenic acid). In contrast, beneficial commensals with significantly decreased abundance in the infected group, such as *Bifidobacterium*, *Blautia*, *Anaerostipes*, and *Agathobacter*, showed significant positive correlations with anti-inflammatory and barrier−protective metabolites (e.g., 11,12-DiHETrE, 6−dehydrotestosterone glucuronide, alpha−linolenic acid) and negative correlations with pro−inflammatory mediators (**Figure 6**).

**Figure 7:**
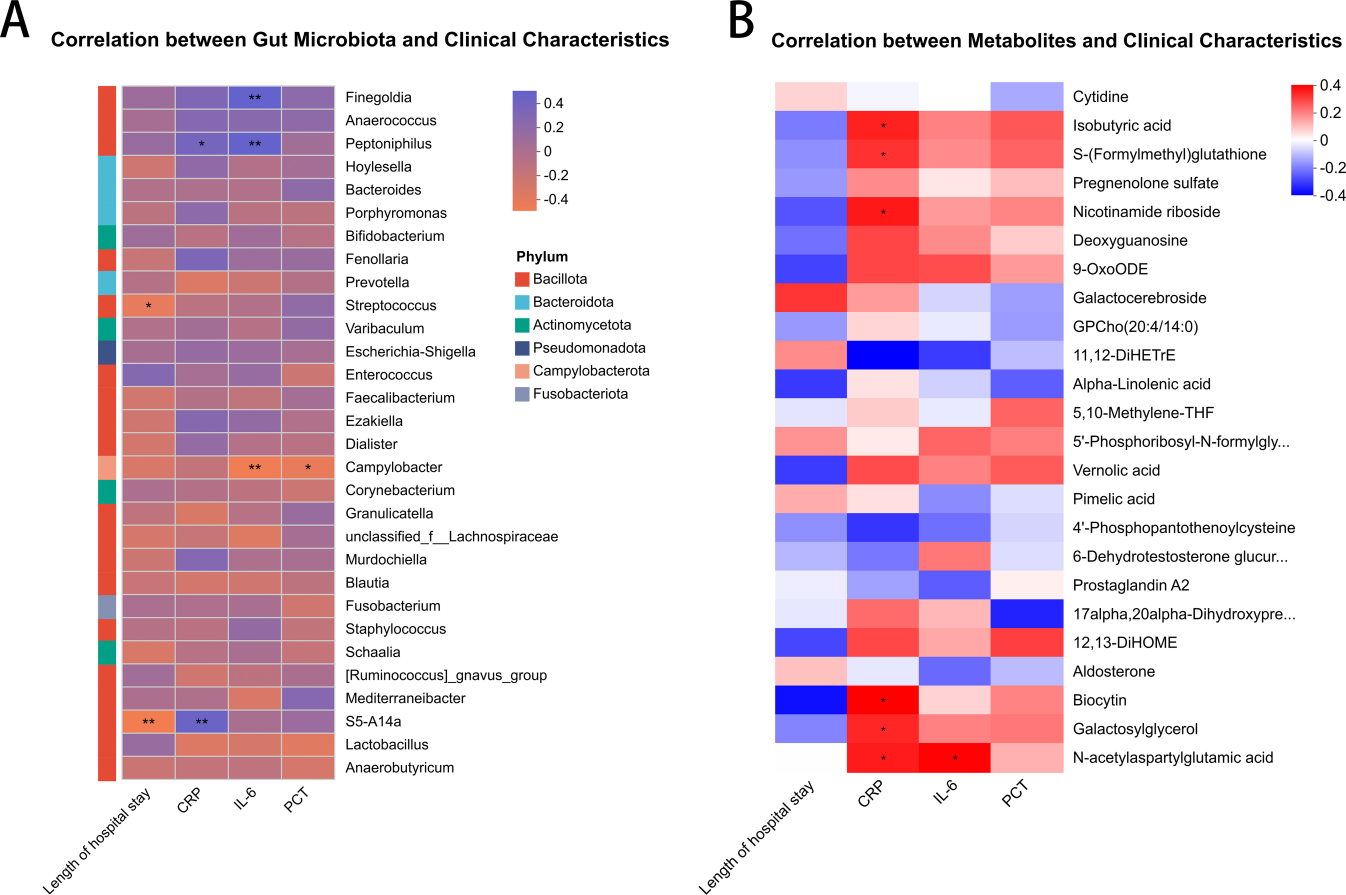
Correlation heatmaps of intestinal genera and metabolites with clinical characteristics. **(A)** Heatmap showing correlations between intestinal genera and length of hospital stay, CRP, IL-6 and PCT; **(B)** Heatmap showing correlations between metabolites and length of hospital stay, CRP, IL-6 and PCT. The color scale represents correlation coefficients, with red indicating positive correlations and blue indicating negative correlations. * 0.01 < P ≤ 0.05, ** 0.001 < P ≤ 0.01, ***P ≤ 0.001. Asterisk-marked cells denote statistically significant correlations.

Spearman’s correlation analysis was performed to evaluate the strength of correlations between gut genera, serum metabolites and four core clinical indicators of children, including length of hospital stay, C-reactive protein (CRP), interleukin-6 (IL-6), and procalcitonin (PCT). The correlation results were visualized in a heatmap (**Figure 7**).

At the genus level (**Figure 7A**), five genera exhibited significant correlations with clinical indicators. Streptococcus (P < 0.05) and S5-A14a (P < 0.01) were significantly negatively correlated with length of hospital stay. Peptoniphilus (P < 0.05) and S5-A14a (P < 0.01) showed significant positive correlations with CRP. Finegoldia (P < 0.01) and Anaerococcus (P < 0.01) were significantly positively correlated with IL-6, whereas Campylobacter (P < 0.01) was significantly negatively correlated with IL-6. In addition, Campylobacter displayed a significant negative correlation with PCT (P < 0.05).

At the metabolite level (**Figure 7B**), most significant correlations were observed with CRP. Isobutyric acid, S-(formylmethyl)glutathione, nicotinamide riboside, biocytin, galactosylglycerol, and N-acetylaspartylglutamic acid were all significantly positively correlated with CRP (P < 0.05). N-acetylaspartylglutamic acid also had a significant positive correlation with IL-6 (P < 0.05). No statistically significant correlations were detected between the tested metabolites and length of hospital stay or PCT.

Collectively, these data revealed that the abundances of genera (Finegoldia, Peptoniphilus, Streptococcus, Campylobacter, S5-A14a) and the concentrations of metabolites (isobutyric acid, S-(formylmethyl)glutathione, nicotinamide riboside, biocytin, galactosylglycerol, N-acetylaspartylglutamic acid) were closely associated with the severity of inflammatory response and hospitalization duration in pediatric patients, which may serve as potential microbiomic biomarkers for evaluating disease severity.

## 4 Discussion

This study is the first to combine 16S rRNA high-throughput sequencing and LC-MS untargeted metabolomics to systematically analyze the gut microbiota characteristics and serum metabolic profiles of children with hMPV infection, providing new experimental evidence for the interaction between respiratory viral infection and the intestinal ecosystem.

In this study, no statistically significant differences were observed in the alpha diversity indices of gut microbiota between the EG and CG groups, which is consistent with findings from studies on RSV infection (29). This observation suggests that hMPV infection may lead to compositional replacement of specific microbiota (e.g., increased abundance of pathogens and decreased commensals) rather than an overall loss of community diversity. Principal component analysis (PCA), principal coordinate analysis (PCoA), and hierarchical clustering consistently revealed a clear separation of microbial community structures between the two groups, indicating that the overall gut microbiota composition of children with hMPV infection deviates from the homeostatic pattern of healthy controls. A significant difference in beta diversity is a core feature of gut dysbiosis, a phenomenon that has been reported in clinical studies of various respiratory pathogens, including influenza virus and SARS-CoV-2 (45, 46). Furthermore, experimental studies using mouse models of influenza infection have also confirmed the disruptive effect of respiratory viral infection on gut microbiota homeostasis (47).

In terms of microbial composition, at the phylum level, the relative abundances of *Bacteroidota* and *Campylobacterota* were significantly increased in the EG group, whereas the abundance of *Actinomycetota* was markedly lower than that in the CG group. The overgrowth of *Bacteroidota* may amplify local inflammatory cascades in synergy with other pathogenic bacteria through the modulation of inflammatory pathways (48). *Campylobacterota* encompasses a range of opportunistic pathogens, and its increased abundance suggests impaired intestinal mucosal barrier and local immune defense (49). *Actinomycetota* includes core beneficial commensals such as *Bifidobacterium*, and its reduced abundance has been consistently documented in various disease−associated gut dysbiosis (50). Collectively, these alterations indicate that children with hMPV infection exhibit a distinct pro−inflammatory phenotype in their gut microbiota.

At the genus level, the EG group showed significant enrichment of *Finegoldia*, *Anaerococcus*, and *Peptoniphilus*. The abnormal proliferation of *Finegoldia* in the intestine may promote the development and progression of inflammation by evading host immune defenses (51), a finding consistent with the gut microbiota profile of patients with invasive pulmonary aspergillosis (52). Elevated abundance of *Anaerococcus* can disrupt gut microbiota homeostasis, a feature also observed in the gut microbiota of patients with SARS-CoV-2 infection (53). In contrast, the EG group exhibited significantly reduced abundances of core short−chain fatty acid (SCFA)-producing genera, including *Blautia*, *Faecalibacterium*, and *Anaerobutyrium* (54). SCFAs help maintain intestinal epithelial barrier integrity and exert systemic anti−inflammatory and immunomodulatory effects. The reduction in these SCFA producers may lead to decreased intestinal SCFA production, thereby weakening their regulatory roles in gut barrier function and systemic inflammation. In the CG group, Bifidobacterium was highly enriched. Its intestinal abundance is significantly negatively correlated with the risk and severity of childhood respiratory tract infections (55, 56). The potential regulatory mechanism underlying these gut microbiota alterations may involve the bidirectional modulation of the “gut−lung axis”. Pulmonary hMPV infection can trigger a strong systemic inflammatory response, leading to substantial release of various pro−inflammatory cytokines. These mediators reach the intestinal microenvironment via the bloodstream, thereby inducing compositional changes in the gut microbiota. Furthermore, the systemic immune response triggered by viral infection, along with reduced appetite and altered dietary intake in affected children, may indirectly affect gut microbiota composition and metabolic profiles (57).

Metabolomics results revealed a global difference in the serum metabolic status of children with hMPV infection. Lipids constituted the largest category of differential metabolites (34.97%), suggesting that lipid metabolism disorder is a core feature of host metabolic abnormalities. Pathway enrichment analysis showed significant enrichment of the glycerophospholipid metabolism pathway, with decreased levels of LPC 16:1, LPC 18:3, and PC 20:1/14/0, and markedly increased levels of oxidized lipids such as 9−OxoODE and 12,13−DiHOME. The downregulation of glycerophospholipids suggests that hMPV infection may disrupt the mucosal barrier, a finding highly consistent with metabolomic studies in patients with acute exacerbation of chronic obstructive pulmonary disease (58). 9-OxoODE is a core marker of lipid peroxidation and oxidative stress (59), and the concomitant upregulation of both 12,13−DiHOME and 9−OxoODE implies that oxidized lipid−mediated inflammation and oxidative stress may be involved in the pathogenesis of hMPV infection. In addition, serum alpha−linolenic acid levels were significantly decreased, and the alpha−linolenic acid metabolism pathway was markedly enriched, possibly reflecting the substantial consumption of host lipid substrates for viral replication and the infection−induced host metabolic reprogramming. This may be an indirect consequence of the virus meeting its own demands for envelope synthesis and replication (60).

Pathway enrichment analysis revealed significant enrichment of linoleic acid metabolism, nucleotide metabolism, and glycerophospholipid metabolism, a pattern consistent with findings from studies on influenza virus infection (61). hMPV is an enveloped, single−stranded, negative−sense RNA virus whose genome replication entirely depends on the host cell for nucleotide precursors. In the present study, serum levels of nucleosides such as cytidine and deoxyguanosine were upregulated in the infected group, while intermediates of the purine synthesis pathway accumulated. These observations likely reflect the broad impact of hMPV infection on host nucleotide metabolism.

The correlation results suggest that hMPV infection may indirectly affect the gut microbiota through systemic inflammation, and that the resulting dysbiosis may in turn amplify lipid metabolic disturbances and inflammatory responses, forming a potential interactive loop. However, this hypothesis requires validation through longitudinal cohort studies.

### Limitation

The following limitations should be considered when interpreting the findings of this study.

First, sample size imbalance. The sample sizes between the case and control groups were imbalanced (42 vs. 12). Post−hoc power analysis revealed that the statistical power to detect a medium effect size (d = 0.5) was only 0.32, suggesting that some negative findings (e.g., alpha diversity indices) should be interpreted with caution, as they may be attributable to insufficient statistical sensitivity. However, the major positive findings (e.g., the genus Bifidobacterium) exhibited a large effect size (d = 1.23) and remained robust under permutation tests and cross−validation.

Second, incomplete control of confounding factors. Although we controlled for core confounders including antibiotic/probiotic use, chronic diseases, and immune status through rigorous exclusion criteria, we did not record dietary habits, breastfeeding history, mode of delivery, or use of non−antibiotic medications. In addition, age was not adjusted for as a covariate in the PERMANOVA models, and although the age distribution was comparable between the two groups, the potential impact of age on microbial developmental trajectories cannot be completely ruled out.

Third, inherent methodological limitations. LEfSe analysis is sensitive to compositional bias and data sparsity; therefore, the differentially abundant taxa identified should be regarded as exploratory and warrant further validation by complementary methods such as qPCR or metagenomic sequencing. Furthermore, metabolite identification was based on MSI Level 2 (putative annotation by MS/MS spectral matching) rather than confirmed by authentic standards, which limits the confidence level of metabolite assignment.

Fourth, missing clinical information. Some patients in the infection group presented with gastrointestinal symptoms, the independent effect of which on gut microbiota cannot be completely excluded. The control group did not undergo nasopharyngeal PCR testing; although active infection was ruled out based on clinical presentation and routine blood tests, the possibility of asymptomatic infection cannot be entirely dismissed. Moreover, clinical indicators such as viral load, pneumonia severity scores, and oxygen saturation were not included in the analysis, limiting our ability to assess the associations between microbiota/metabolites and disease severity.

In summary, the findings of this study are exploratory and hypothesis−generating, and require further validation through larger, prospective, multicenter cohorts and mechanistic experiments.

## 5 Conclusion

This study found that children infected with human metapneumovirus (hMPV) present intestinal microecological imbalance, which is characterized by reduced abundances of beneficial commensals including Bifidobacterium, Blautia, and Faecalibacterium, as well as enriched opportunistic pathogens represented by Finegoldia, Anaerococcus, and Hoylesella. Meanwhile, children with hMPV infection exhibit significant alterations in serum metabolic profiles. Multiple metabolic pathways are markedly disturbed in infected children, covering glycerophospholipid metabolism, nucleotide metabolism, alpha-linolenic acid metabolism, and arachidonic acid metabolism. The core differential metabolites screened in this study exert excellent diagnostic performance for pediatric hMPV infection and can be exploited as potential biomarkers. Moreover, tight correlations were confirmed between gut microbiota dysbiosis and serum metabolic disorders in hMPV-infected children. The depletion of beneficial commensals is closely associated with the downregulation of metabolites previously reported to be involved in anti-inflammatory and barrier-protective functions, whereas the proliferation of opportunistic pathogens is accompanied by the accumulation of lipid mediators recognized to be correlated with inflammatory processes. Additionally, several differential metabolites identified in the present study have been documented to be associated with oxidative stress pathways in existing literature. The above findings suggest that targeting gut microbiota and host metabolic pathways may provide a novel research direction for the prevention and treatment of pediatric hMPV infection. Nevertheless, the clinical translational value of these targeted strategies needs to be further verified by large-sample prospective studies and mechanistic experiments.

## Data Availability

The original contributions presented in the study are publicly available. This data can be found here: http://www.ncbi.nlm.nih.gov/bioproject/1496541 (BioProject ID: PRJNA1496541).

## Supplementary material

Supplementary Figure 1 Rarefaction curve analysis of gut microbiota in hMPV-infected children (EG) and healthy controls (CG). **(A)** Shannon index curves showing bacterial community diversity; **(B)** Sobs index curves reflecting bacterial community richness.

Supplementary Figure 2 Gut microbiota composition at the phylum level in hMPV-infected (EG) and healthy control (CG) children. **(A)** Community barplot analysis; **(B, C)** Pie plots of phylum-level distribution; **(D)** Wilcoxon rank-sum test bar plot showing differential phyla.

Supplementary Table 1 Clinical characteristics of 42 children with hMPV infection.

Supplementary Table 2 Laboratory findings of children with hMPV infection.

Supplementary Table 3 Summary of valid sequence counts and ASV numbers for each sample.

Supplementary Table 4 List of differential metabolites with VIP > 1.5 in children with hMPV infection.

Supplementary Table 5 Diagnostic performance of key serum metabolites for pediatric hMPV infection.

Supplementary Table 6 Metadata of 24 Key Differential Metabolites (FDR Correction Included).

Supplementary Table 7 Software and Versions Used for 16S rRNA Sequencing Data Analysis.

Supplementary Table 8 **Supplementary Table 8** Software and Versions for Metabolomic Data Analysis.
