# Supplementary material for: Gut microbiota and serum metabolome in children with human metapneumovirus infection
Source: Front Immunol. 2026 Jul 28;17:1855983. doi: 10.3389/fimmu.2026.1855983 (PMC13459443; doi:10.3389/fimmu.2026.1855983)
Supplement: Supplementary file 1 [file DataSheet1.docx]

# Supplementary Material

**Supplementary figures**

**Supplementary Figure 1.**Rarefaction curve analysis of gut microbiota in hMPV-infected children (EG) and healthy controls (CG). (A) Shannon index curves showing bacterial community diversity; (B) Sobs index curves reflecting bacterial community richness.

**Supplementary Figure 2.**Gut microbiota composition at the phylum level in hMPV-infected (EG) and healthy control (CG) children. (A) Community barplot analysis; (B, C) Pie plots of phylum-level distribution; (D) Wilcoxon rank-sum test bar plot showing differential phyla.

**Supplementary table captions**

**Supplementary Table 1.** Clinical characteristics of 42 children with hMPV infection.

**Supplementary Table 2.**Laboratory findings of children with hMPV infection.

**Supplementary Table 3.** Summary of valid sequence counts and ASV numbers for each sample.

**Supplementary Table 4.** List of differential metabolites with VIP > 1.5 in children with hMPV infection.

**Supplementary Table 5.** Diagnostic performance of key serum metabolites for pediatric hMPV infection.

**Supplementary Table 6.** Metadata of 24 Key Differential Metabolites (FDR Correction Included).

**Supplementary Table 7.** Software and Versions Used for 16S rRNA Sequencing Data Analysis.

**Supplementary Table 8.** Supplementary Table 8 Software and Versions for Metabolomic Data Analysis.

**Supplementary figures**

**Supplementary Figure 1**

**Supplementary Figure 2**

**Supplementary table captions**

**Supplementary Table 1**

|  | Number of positive cases (n) | Percentage (%) |
| --- | --- | --- |
| **Gender** |  |  |
| Male | 20 | 47.62 |
| Female | 22 | 52.38 |
| **Age group** |  |  |
| ≥29 days to ≤3 years | 22 | 52.38 |
| >3 years to ≤5 years | 13 | 30.95 |
| >5 years | 7 | 16.68 |
| **Clinical symptoms** |  |  |
| Fever | 42 | 100 |
| Cough | 42 | 100 |
| Rhinorrhea | 36 | 85.71 |
| Sputum production | 33 | 78.57 |
| Wheezing | 4 | 9.52 |
| Tachypnea | 2 | 4.76 |
| Cyanosis | 1 | 2.38 |
| Gastrointestinal symptoms | 12 | 28.57 |
| **Pulmonary signs** |  |  |
| Abnormal rales | 32 | 76.19% |
| Isolated moist rales | 17 | 53.13 |
| Isolated dry rales | 1 | 3.13 |
| Combined moist and dry rales | 14 | 43.75 |
| **Imaging findings** |  |  |
| Bronchitis | 4 | 9.52 |
| Pneumonia | 38 | 90.48 |
| **Length of hospital stay** |  |  |
| <3 days | 4 | 9.52 |
| ≥3 days to ≤5 days | 33 | 78.57 |
| >5 days | 5 | 11.9 |

**Supplementary Table 2**

| Item | Measured value P50 (P25, P75) | Increased (n) | Decreased (n) |
| --- | --- | --- | --- |
| White blood cell (WBC) count | 7.77（5.38，11.32） | 7 | 7 |
| Hemoglobin concentration | 122（112，126） | 0 | 7 |
| Platelet count | 269（232，353） | 1 | 5 |
| Neutrophil count | 3.25（1.97，5.44） | 8 | 4 |
| Lymphocyte count | 3（1.75，4.29） | 2 | 10 |
| C-reactive protein (CRP) | 1.58（4.7，13.84） | 11 | 0 |
| Procalcitonin (PCT) | 0.194（0.081，0.392） | 40 | 0 |
| Interleukin-6 (IL-6) | 19.01（8.13，49.11） | 34 | 0 |

**Supplementary Table 3**

| Sample ID | Number of ASVs | Effective reads | Sample ID | Number of ASVs | Effective reads数 |
| --- | --- | --- | --- | --- | --- |
| A01 | 114 | 20590 | A28 | 67 | 20590 |
| A02 | 22 | 20590 | A29 | 64 | 20590 |
| A03 | 207 | 20590 | A31 | 99 | 20590 |
| A04 | 127 | 20590 | A32 | 158 | 20590 |
| A05 | 168 | 20590 | A33 | 119 | 20590 |
| A06 | 125 | 20590 | A34 | 70 | 20590 |
| A07 | 88 | 20590 | A35 | 126 | 20590 |
| A08 | 193 | 20590 | A36 | 115 | 20590 |
| A09 | 102 | 20590 | A37 | 137 | 20590 |
| A10 | 92 | 20590 | A38 | 177 | 20590 |
| A11 | 94 | 20590 | A39 | 39 | 20590 |
| A12 | 25 | 20590 | A40 | 138 | 20590 |
| A13 | 145 | 20590 | A41 | 126 | 20590 |
| A14 | 56 | 20590 | A42 | 40 | 20590 |
| A15 | 80 | 20590 | A43 | 83 | 20590 |
| A16 | 182 | 20590 | B01 | 69 | 20590 |
| A17 | 85 | 20590 | B02 | 97 | 20590 |
| A18 | 189 | 20590 | B03 | 135 | 20590 |
| A19 | 257 | 20590 | B04 | 96 | 20590 |
| A20 | 49 | 20590 | B05 | 84 | 20590 |
| A21 | 61 | 20590 | B06 | 58 | 20590 |
| A22 | 171 | 20590 | B07 | 168 | 20590 |
| A23 | 94 | 20590 | B08 | 138 | 20590 |
| A24 | 201 | 20590 | B09 | 86 | 20590 |
| A25 | 141 | 20590 | B10 | 122 | 20590 |
| A26 | 86 | 20590 | B11 | 60 | 20590 |
| A27 | 117 | 20590 | B12 | 79 | 20590 |

**Supplementary Table 4**

| Metabolite | VIP | FC | Regulate |
| --- | --- | --- | --- |
| Galactosylglycerol | 2.6802 | 2.0267 | up |
| S-(Formylmethyl)glutathione | 2.6624 | 1.531 | up |
| Vernolic acid | 2.5232 | 1.3587 | up |
| Biocytin | 2.4956 | 1.6926 | up |
| Isobutyric acid | 2.3819 | 1.4321 | up |
| Pregnenolone sulfate | 2.38 | 2.1524 | up |
| 9-OxoODE | 2.2837 | 1.2792 | up |
| 6-Dehydrotestosterone glucuronide | 2.1592 | 0.4804 | down |
| 4'-Phosphopantothenoylcysteine | 2.0392 | 0.745 | down |
| Prostaglandin A2 | 1.9117 | 1.7839 | up |
| Alpha-Linolenic acid | 1.8819 | 0.8052 | down |
| 12,13-DiHOME | 1.8664 | 1.1473 | up |
| 5,10-Methylene-THF | 1.8508 | 1.2806 | up |
| Nicotinamide riboside | 1.7677 | 1.2938 | up |
| 17alpha,20alpha-Dihydroxypregn-4-en-3-one | 1.7637 | 1.8346 | up |
| 5'-Phosphoribosyl-N-formylglycinamidine | 1.7103 | 1.359 | up |
| Cytidine | 1.6754 | 1.4011 | up |
| Aldosterone | 1.6458 | 1.3103 | up |
| Galactocerebroside | 1.6029 | 1.1318 | up |
| N-acetylaspartylglutamic acid | 1.5983 | 1.2309 | up |
| Deoxyguanosine | 1.5478 | 1.2468 | up |
| 11,12-DiHETrE | 1.5428 | 0.8786 | down |
| GPCho(20:4/14:0) | 1.503 | 0.8584 | down |
| Pimelic acid | 1.501 | 1.3166 | up |

**Supplementary Table 5**

| Metabolite | AUC | CI |
| --- | --- | --- |
| N-acetylaspartylglutamic acid | 1 | [0.8283, 1] |
| 5'-Phosphoribosyl-N-formylglycinamidine | 0.91 | [0.7026, 1] |
| S-(Formylmethyl)glutathione | 1 | [0.9167, 1] |
| Vernolic acid | 1 | [0.8889, 1] |
| 12,13-DiHOME | 1 | [0.8611, 1] |
| Aldosterone | 0.36 | [0.0714, 1] |
| 9-OxoODE | 1 | [0.92, 1] |
| 5,10-Methylene-THF | 1 | [0.8636, 1] |
| Pregnenolone sulfate | 1 | [0.9091, 1] |
| Isobutyric acid | 1 | [0.8611, 1] |
| Galactosylglycerol | 1 | [0.8611, 1] |
| 6-Dehydrotestosterone glucuronide | 0.57 | [0.183, 1] |
| Cytidine | 1 | [0.8077, 1] |
| 17alpha,20alpha-Dihydroxypregn-4-en-3-one | 0.68 | [0.3846, 1] |
| Alpha-Linolenic acid | 0.89 | [0.3846, 1] |
| Prostaglandin A2 | 0.95 | [0.7176, 1] |
| Galactocerebroside | 1 | [0.8182, 1] |
| Nicotinamide riboside | 1 | [0.7857, 1] |
| 4'-Phosphopantothenoylcysteine | 0.91 | [0.6956, 1] |
| Biocytin | 1 | [0.8611, 1] |
| Pimelic acid | 0.48 | [0.1923, 1] |
| Deoxyguanosine | 1 | [0.7913, 1] |
| GPCho(20:4/14:0) | 0.95 | [0.72, 1] |
| 11,12-DiHETrE | 0.95 | [0.7857, 1] |

**Supplementary Table 6**

| Metabolite | P_value | FDR | VIP_pred_OPLS-DA | VIP_PLS-DA | FC(HX/YY) | Log2FC(HX/YY) |
| --- | --- | --- | --- | --- | --- | --- |
| S-(Formylmethyl)glutathione | 1.105E-19 | 4.219E-17 | 2.6624 | 2.4596 | 1.531 | 0.6145 |
| Vernolic acid | 4.66E-18 | 9.866E-16 | 2.5232 | 2.3335 | 1.3587 | 0.4423 |
| 9-OxoODE | 1.421E-17 | 2.563E-15 | 2.2837 | 2.1102 | 1.2792 | 0.3553 |
| Isobutyric acid | 5.036E-16 | 5.245E-14 | 2.3819 | 2.1838 | 1.4321 | 0.5182 |
| 12,13-DiHOME | 1.056E-15 | 9.561E-14 | 1.8664 | 1.7315 | 1.1473 | 0.1983 |
| Galactosylglycerol | 1.267E-13 | 6.624E-12 | 2.6802 | 2.4416 | 2.0267 | 1.0192 |
| Pregnenolone sulfate | 2.204E-13 | 1.095E-11 | 2.38 | 2.222 | 2.1524 | 1.1059 |
| Biocytin | 8.247E-13 | 3.625E-11 | 2.4956 | 2.2587 | 1.6926 | 0.7593 |
| Galactocerebroside | 1.314E-12 | 5.4E-11 | 1.6029 | 1.5247 | 1.1318 | 0.1787 |
| 5,10-Methylene-THF | 8.488E-12 | 2.821E-10 | 1.8508 | 1.7478 | 1.2806 | 0.3569 |
| N-acetylaspartylglutamic acid | 2.373E-8 | 2.31E-7 | 1.5983 | 1.6521 | 1.2309 | 0.2997 |
| Nicotinamide riboside | 2.821E-8 | 2.669E-7 | 1.7677 | 1.6184 | 1.2938 | 0.3716 |
| 11,12-DiHETrE | 4.545E-8 | 3.974E-7 | 1.5428 | 1.4857 | 0.8786 | -0.1868 |
| Deoxyguanosine | 3.421E-7 | 2.188E-6 | 1.5478 | 1.4118 | 1.2468 | 0.3183 |
| Cytidine | 6.948E-7 | 3.995E-6 | 1.6754 | 1.5981 | 1.4011 | 0.4866 |
| 4'-Phosphopantothenoylcysteine | 1.984E-6 | 9.765E-6 | 2.0392 | 1.8788 | 0.745 | -0.4247 |
| Alpha-Linolenic acid | 4.683E-6 | 2.065E-5 | 1.8819 | 1.8683 | 0.8052 | -0.3125 |
| 5'-Phosphoribosyl-N-formylglycinamidine | 8.159E-6 | 3.394E-5 | 1.7103 | 1.5503 | 1.359 | 0.4426 |
| GPCho(20:4/14:0) | 2.031E-5 | 7.777E-5 | 1.4999 | 1.4312 | 0.8584 | -0.2202 |
| Pimelic acid | 2.75E-5 | 0.0001023 | 1.4938 | 1.6269 | 1.3166 | 0.3968 |
| Prostaglandin A2 | 3.28E-5 | 0.0001197 | 1.9117 | 1.8816 | 1.7839 | 0.835 |
| Aldosterone | 0.0002452 | 0.00074 | 1.6458 | 1.9269 | 1.3103 | 0.3899 |
| 6-Dehydrotestosterone glucuronide | 0.0004515 | 0.00128 | 2.1592 | 2.1211 | 0.4804 | -1.0577 |
| 17alpha,20alpha-Dihydroxypregn-4-en-3-one | 0.004681 | 0.01033 | 1.7637 | 2.2987 | 1.8346 | 0.8755 |

**Supplementary Table 7**

| Analysis Software / Database | Version | Database Link |
| --- | --- | --- |
| fastp | 0.23.4 | https://github.com/OpenGene/fastp |
| FLASH | 1.2.11 | https://ccb.jhu.edu/software/FLASH/index.shtml |
| Qiime2 | 2024 | https://qiime2.org |
| DADA2 | 2024 | https://qiime2.org |
| Deblur | 2024 | https://benjjneb.github.io/dada2/ |
| Mega | 7 | https://www.megasoftware.net/ |
| SILVA | 138 | https://www.arb-silva.de/ |
| UNITE | 8 | https://unite.ut.ee/ |
| RDP | 11.5 | http://rdp.cme.msu.edu/ |
| GreenGenes | 135 | http://greengenes.secondgenome.com/ |
| FunGene | 9.6 | http://www.fungene-db.fr/ |
| MaarjAM | 81 | https://www.maarjam.botany.ut.ee/ |
| HPB | none | https://www.cerl.org/resources/hpb/content |
| PR2 | 1 | https://github.com/vaulot/pr2_database |
| Funguild | 1 | http://www.funguild.org/ |
| Tax4fun | 0.3.1 | http://tax4fun.gobics.de/ |
| MAFFT | 7.2 | https://mafft.cbrc.jp/alignment/software/ |
| IQ-TREE | 1.6.8 | http://www.iqtree.org/ |
| PICRUSt2 | 2.2.0 | https://github.com/picrust/picrust2/ |
| Mothur | 1.48.3 | none |
| PICRUSt | 1.1.0 | http://picrust.github.io/picrust/ |

**Supplementary Table 8**

| Analysis Item | Software | Software Version | Software Parameters |
| --- | --- | --- | --- |
| Expression data preprocessing | Majorbio self-developed software | none | None |
| PCA analysis | ropls (R package) | Version1.6.2 | Default parameters |
| Differential metabolite analysis 每 multivariate statistics | ropls (R package) | Version1.6.2 | Default parameters |
| KEGG compound classification | KEGG (kegg_v20241007) | Release 2017-05-01 | None |
| KEGG functional pathways | KEGG (kegg_v20241007) | Release 2017-05-01 | None |
| Venn diagram | VennDiagram (R package) | Version1.6.20 | None |
| KEGG pathway enrichment analysis | scipy (Python) | Version1.0.0 | p-value < 0.05 |
| Heatmap | scipy (Python) | Version1.0.0 | None |
| Cluster analysis | scipy (Python) | Version1.0.0 | None |
| Correlation analysis | scipy (Python) | Version1.0.0 | None |
| VIP analysis | ropls (R); scipy (Python) | Version1.6.2;Version1.0.0 | None |
| iPath metabolic pathway analysis | iPath 3.0 | Version3.0 | None |
| ROC curve analysis | pROC (R package) | Version1.12.1 | None |
